# Increased Resting-State Cerebellar-Cerebral Functional Connectivity Underlying Chronic Tinnitus

**DOI:** 10.3389/fnagi.2018.00059

**Published:** 2018-03-05

**Authors:** Yuan Feng, Yu-Chen Chen, Han Lv, Wenqing Xia, Cun-Nan Mao, Fan Bo, Huiyou Chen, Jin-Jing Xu, Xindao Yin

**Affiliations:** ^1^Department of Radiology, Nanjing First Hospital, Nanjing Medical University, Nanjing, China; ^2^Department of Radiology, Beijing Friendship Hospital, Capital Medical University, Beijing, China; ^3^Department of Endocrinology, Nanjing First Hospital, Nanjing Medical University, Nanjing, China; ^4^Department of Otolaryngology, Nanjing First Hospital, Nanjing Medical University, Nanjing, China

**Keywords:** tinnitus, cerebellum, functional connectivity, resting-state fMRI

## Abstract

**Purpose**: Chronic subjective tinnitus may arise from aberrant functional coupling between the cerebellum and the cerebral cortex. To explore this hypothesis, we used resting-state functional magnetic resonance imaging (fMRI) to illuminate the functional connectivity network of the cerebellar regions in chronic tinnitus patients and controls.

**Methods**: Resting-state fMRI scans were obtained from 28 chronic tinnitus patients and 29 healthy controls (well matched for age, sex and education) in this study. Cerebellar-cerebral functional connectivity was characterized using a seed-based whole-brain correlation method. The resulting cerebellar functional connectivity measures were correlated with each clinical tinnitus characteristic.

**Results**: Chronic tinnitus patients demonstrated increased functional connectivity between the cerebellum and several cerebral regions, including the superior temporal gyrus (STG), parahippocampal gyrus (PHG), inferior occipital gyrus (IOG), and precentral gyrus. The enhanced functional connectivity between the left cerebellar Lobule VIIb and the right STG was positively correlated with the Tinnitus Handicap Questionnaires (THQ) score (*r* = 0.577, *p* = 0.004). Furthermore, the increased functional connectivity between the cerebellar vermis and the right STG was also associated with the THQ score (*r* = 0.432, *p* = 0.039).

**Conclusions**: Chronic tinnitus patients have greater cerebellar functional connectivity to certain cerebral brain regions which is associated with specific tinnitus characteristics. Resting-state cerebellar-cerebral functional connectivity disturbances may play a pivotal role in neuropathological features of tinnitus.

## Introduction

Tinnitus is defined as a phantom auditory perception, such as ringing, roaring, or buzzing in ears without any external sounds (Jastreboff, 1990; Lockwood et al., 2002; Wegger et al., 2017). The prevalence of tinnitus has been reported to be approximately 10% to 15% of adults in the United States (Henry et al., 2005; Hall et al., 2011; Langguth et al., 2013). Insomnia, depression and anxiety which often significantly impair the quality of daily life can often be found in patients with chronic tinnitus (Reynolds et al., 2004; Langguth et al., 2013). Prior studies found that the central nervous system may play a major role in the pathophysiology of tinnitus (Lockwood et al., 2002; Eggermont, 2005; Bartels et al., 2007; Chen et al., 2015a). Tinnitus may be derived from aberrant neural activity in the central auditory pathway or in the acoustic center other than the cochlea, as shown in previous electrophysiological and neuroimaging studies (Lockwood et al., 1998; Kaltenbach et al., 2005). After auditory nerve transection, the phantom experience of tinnitus still does not disappear (Jackler and Whinney, 2001). It has been demonstrated that tinnitus involves aberrant neural activity in not only auditory regions but also non-auditory structures such as the prefrontal cortex, parahippocampal gyrus (PHG), amygdala, and cerebellum, which have been confirmed to be important in the development or progression of tinnitus (Rauschecker et al., 2010; Leaver et al., 2011; Langguth et al., 2013; Chen et al., 2015a, 2017a,b,c; Brozoski et al., 2017).

The cerebellum plays an essential role in complicated circuits of sensorimotor, autonomic and cognitive functioning (Baumann et al., 2015). The connections between the cerebellum and the cerebral cortex are composed of feedforward (the corticopontine-pontocerebellar circuit) and feedback (the cerebellothalamic-thalamocortical circuit) loops, which are the anatomic foundations of the involvement of the cerebellum in sensory perception (Baumann et al., 2015). The cerebellum is structurally connected to the cochlear nucleus (Huang et al., 1982), superior olivary nucleus (Rossi et al., 1967), inferior colliculus (Ruchalski and Hathout, 2012), medial geniculate body (Keifer et al., 2015) and the auditory cortex (Huffman and Henson, 1990), either directly or indirectly. This suggests that the cerebellum could impact the signal from the peripheral hearing organs or modulate the activity of the acoustic center. Previous studies demonstrated that the unipolar brush cells upregulate and enhance glutamatergic transmission in the cerebellum and contribute to the pathophysiology of tinnitus (Bauer et al., 2013b; Brozoski et al., 2017). Moreover, the paraflocculus lobe of the cerebellum, which integrates the information from the vestibular and auditory centers, has been confirmed to be involved in the modulation of tinnitus by examination of electrophysiological changes (Chen et al., 2017a). Shulman et al. (1995) first found increased regional cerebral blood flow (rCBF) in the cerebellum of severe tinnitus patients using single photon emission computed tomography (SPECT). Ueyama et al. (2015) demonstrated that the rCBF in tinnitus patients was significantly higher in the cerebellar hemispheres and vermis. Using positron emission tomography (PET), Petacchi et al. (2005, 2011) have specifically tested for a pure sensory role for the cerebellum in auditory processing.

The cerebellum showed a significantly larger response in the modulated networks of tinnitus patients compared to those of controls using sound stimuli-based functional magnetic resonance imaging (fMRI; Boyen et al., 2014; Lanting et al., 2014). Moreover, resting-state fMRI of spontaneous blood oxygenation level-dependent (BOLD) responses has proved to be a useful noninvasive technique to evaluate the potential neural pathogenesis underlying tinnitus (Husain and Schmidt, 2014; Chen et al., 2017b). Using resting-state fMRI, Chen et al. (2015a) investigated hyperactivity in the cerebellum and increased functional connectivity between the auditory cortex and the cerebellum in rats with salicylate-induced tinnitus. To date, cerebellar-cerebral functional connectivity has not been systematically explored in human tinnitus patients.

Given the crucial role that the cerebellum appears to play in the neuropathology of tinnitus, a seed-based approach was used to investigate differences in cerebellar resting-state functional connectivity between tinnitus patients and healthy controls. We hypothesized that resting-state cerebellar-cerebral functional connectivity in tinnitus patients would be significantly distinct from that in controls and that some of the aberrant functional connectivity would be correlated with specific tinnitus characteristics such as the severity of tinnitus distress.

## Materials and Methods

### Subjects

**Figure 1:**
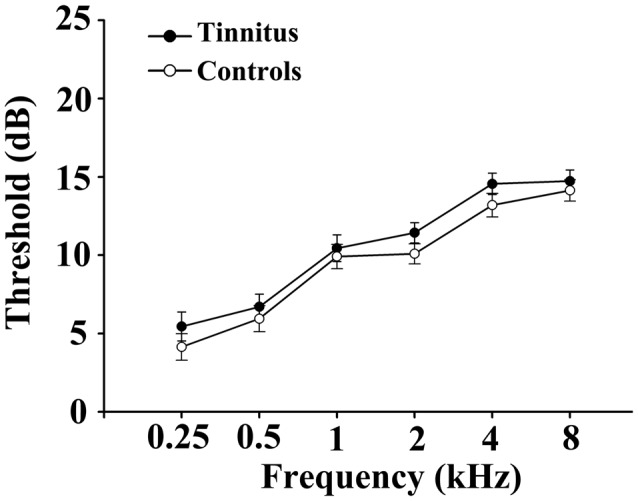
No significant differences in the auditory thresholds between the tinnitus and control groups. Data are presented as mean ± SEM.

This study included 28 chronic tinnitus patients and 29 healthy subjects (all right handed, with at least 8 years of education). The tinnitus subjects were outpatients at the clinic of the Department of Otolaryngology at the Nanjing First Hospital. The healthy controls were recruited through community health screening or newspaper advertisements. None of the participants were excluded because of exceeded limits for head motion during scanning. The groups were matched in terms of age, sex and education. Thirteen patients reported a predominantly left-sided tinnitus, seven a predominantly right-sided tinnitus and eight described their tinnitus as bilateral or originating within the head. The severity of the tinnitus and related distress were assessed by the Iowa version of the Tinnitus Handicap Questionnaires (THQ; Kuk et al., 1990). Based on the THQ score and previously proposed guidelines (McCombe et al., 2001), the severity of the tinnitus patients was categorized as mild, moderate or severe. Seven patients had mild tinnitus, 17 had moderate tinnitus, and four had severe tinnitus in this study. The hearing threshold was determined by a puretone audiometry (PTA) examination. None of the participants had hearing loss in any of six measured audiometric frequencies ranging from 250 Hz to 8 kHz (hearing thresholds <25 dB). There were no significant differences in the auditory thresholds between the tinnitus and control groups (Figure 1 and Table 1). No included participants had accompanying symptoms of depression or anxiety according to the Self-Rating Depression Scale (SDS) and the Self-Rating Anxiety Scale (SAS; overall scores <50), respectively (Zung, 1971, 1986). According to a previous study (Khalfa et al., 2002), we used the Hyperacusis Questionnaire to exclude participants with hyperacusis from the current study. Participants were also excluded if they suffered from Meniere’s disease, pulsatile tinnitus or hyperacusis, or if they had a history of severe alcoholism, smoking, head injury, stroke, Alzheimer’s disease, Parkinson’s disease, epilepsy, major depression, other neurological or psychiatric illness, major medical illnesses (e.g., cancer, anemia and thyroid dysfunction), MRI contraindications, and/or severe vision loss. Table 1 summarizes the characteristics of the chronic tinnitus patients and the healthy subjects. All the participants provided written informed consent before their participation in the study protocol, which was approved by the Research Ethics Committee of Nanjing Medical University (Reference No. 2016067).

**Table 1:** Characteristics of tinnitus patients and healthy controls.

	Tinnitus patients (*n* = 28)	Healthy controls (*n* = 29)	*p* value
Age (years)	50.2 ± 12.8	44.3 ± 14.6	0.114
Gender (male: female)	9:19	10:19	0.851
Education levels (years)	12.6 ± 2.9	13.3 ± 3.0	0.349
Tinnitus duration (months)	47.8 ± 40.0	−	−
THQ score	50.8 ± 16.3	−	−
Gray matter	581.18 ± 26.88	575.55 ± 21.72	0.388
White matter	533.71 ± 24.95	525.52 ± 25.13	0.222
Brain parenchyma	1114.89 ± 33.43	1101.07 ± 37.90	0.150
Hearing thresholds (left)	15.0 ± 3.0	14.1 ± 2.6	0.221
Hearing thresholds (right)	16.1 ± 2.6	15.1 ± 2.7	0.146
Hearing thresholds (average)	15.6 ± 2.1	14.6 ± 1.9	0.066
FD value	0.21 ± 0.07	0.20 ± 0.06	0.362

*Data are represented as mean ± SD. THQ, Tinnitus Handicap Questionnaire; FD, framewise displacement*.

### MR Scanning

All subjects were scanned using a 3.0 T MRI scanner (Ingenia, Philips Medical Systems, Netherlands) with a standard head coil. Head motion and scanner noise were reduced using foam padding and earplugs. The earplugs (Hearos Ultimate Softness Series, USA) were used to attenuate scanner noise by approximately 32 dB. The subjects were instructed to lie quietly with their eyes closed, but not to fall asleep and to avoid thinking of anything in particular during the scanning. Structural images were acquired with a three-dimensional turbo fast echo (3D-TFE) T1WI sequence with high resolution as follows: repetition time (TR)/echo time (TE) = 8.1/3.7 ms; slices = 170; thickness = 1 mm; gap = 0 mm; flip angle (FA) = 8°; acquisition matrix = 256 × 256; field of view (FOV) = 256 × 256 mm. The structural sequence took 5 min and 29 s. Functional images were obtained axially using a gradient echo-planar imaging sequence as follows: TR = 2000 ms; TE = 30 ms; slices = 36; thickness = 4 mm; gap = 0 mm; FOV = 240 mm × 240 mm; acquisition matrix = 64 × 64; and FA = 90°. The fMRI sequence took 8 min and 8 s.

### Data Preprocessing

Data were preprocessed using Data Processing Assistant for Resting-State fMRI programs (Chao-Gan and Yu-Feng, 2010), which is based on Statistical Parametric Mapping (SPM8^1^) and the resting-state fMRI data analysis toolkit (REST^2^). The first 10 volumes were discarded, and the remaining 230 consecutive volumes were used for data analysis. Slice-timing and realignment for head motion correction were performed. Any subjects with head motion of >2.0 mm translation or 2.0° rotation in any direction were excluded. After that, spatial normalization using T1 image unified segmentation (resampling voxel size = 3 × 3 × 3 mm^3^), smoothing with an isotropic Gaussian kernel (full width at half maximum (FWHM) = 6 mm), detrending and filtering (0.01–0.08 Hz) were performed in order.

### Structural Data Analysis

A voxel-based morphometry (VBM) approach was performed to compute the gray matter (GM) volume and white matter (WM) volume of each subject using the VBM8 toolbox^3^. Briefly, cerebral tissues were segmented into GM, WM and cerebrospinal fluid and were then normalized to the MNI space using a unified segmentation algorithm (Ashburner and Friston, 2005). T1 images were normalized to the MNI template using affine linear registration followed by Gaussian smoothing (FWHM = 6 mm). GM and WM volumes were calculated by estimating these segments. The brain parenchyma volume was calculated as the sum of the GM and WM volumes.

### Functional Connectivity Analysis

Functional connectivity was analyzed using REST software. Nine seed region of interests (ROIs) of the cerebellum were generated using WFU_PickAtlas software (Maldjian et al., 2003), including bilateral Crus I, bilateral Crus II, bilateral Lobule VI, bilateral Lobule VIIb and Vermis. These specific regions of the cerebellum have been documented to participate in higher-order functions such as auditory sensory processing, attentional and emotional functioning (Petacchi et al., 2005; Stoodley and Schmahmann, 2009; Timmann et al., 2010). These ROIs have also been found to contribute to intrinsic functional connectivity networks in healthy controls, such as the default mode network, attention network and salience network (Habas et al., 2009; Krienen and Buckner, 2009; Stoodley and Schmahmann, 2009; Timmann et al., 2010). Since tinnitus is believed to be generated by aberrant neural activity in the central auditory pathway and multiple abnormal functional connectivity networks related to tinnitus have been demonstrated in previous studies (Husain and Schmidt, 2014; Chen et al., 2016; Leaver et al., 2016), we selected the bilateral Lobule VI, bilateral Crus I, bilateral Crus II, bilateral Lobule VIIb and Vermis as the ROIs in this study. The mean time series of each ROI was acquired for reference time course. Pearson’s correlation coefficients were then computed between the mean signal change of each ROI and the time series of each voxel. Finally, the correlation coefficients were converted into *z*-values using Fisher z-transform to improve the normality (Lowe et al., 1998). Six parameters of head motion and the average time courses of the global, WM and CSF signals were removed by linear regression analysis.

For the within-group analysis, each individual’s z map was entered into the SPM8 software for random effect one-sample *t*-tests to determine the brain regions showing significant connectivity to each cerebellar ROI at a threshold of *p* < 0.05 with multiple comparisons correction using the false discovery rate (FDR) criterion. Two-sample *t*-tests were performed to identify differences in functional connectivity of each cerebellar ROI between the tinnitus patients and controls within a default whole-brain mask. Age, sex, education, GM volume and average hearing thresholds were included as nuisance covariates. The significance of group differences was assessed using cluster-level inference at *p* < 0.01 corrected by family-wise error (FWE). Based on the suggestion from the prior study (Eklund et al., 2016), in the current study, we further applied a permutation test (Winkler et al., 2014) which was based on the PALM package in DPABI software (Yan et al., 2016). The permutation number was 5000 and the cluster forming threshold (z) was 2.3. A kind of acceleration method (few permutations) that is embedded in the PALM package was used. This procedure was considered to be valid for any spatial autocorrelation function.

### Statistical Analysis

Between-group *t*-tests and *χ*^2^-tests were used to analyze the differences in the demographic data between the tinnitus patients and healthy controls (*p* < 0.05 was considered to be significant). To investigate the relationship between fMRI data and clinical characteristics of the tinnitus patients, regions showing significant increased functional connectivity between groups were extracted. Then, the mean *z-*values within these clusters were correlated against each clinical characteristic of tinnitus patients using Pearson’s correlation analysis by SPSS software (version 19.0; SPSS, Chicago, IL, USA). Statistical threshold was set at *p* < 0.05. Partial correlations were calculated after correction for age, sex, education, GM volume and average hearing thresholds. Bonferroni correction for multiple comparisons was applied in the correlation analyses.

Since micromovements from volume to volume can influence the functional connectivity (Power et al., 2012), framewise displacement (FD) values were computed for each subject to reflect the temporal derivative of the movement parameters. No subjects had FD >0.5 mm on more than 35 volumes in this study. No significant difference was found in the mean FD values between the tinnitus patients and controls (Table 1).

## Results

### Structural Analysis

Comparisons of the whole brain volumes (GM volume, WM volume and brain parenchyma volume) between the tinnitus patients and healthy subjects are presented in Table 1. No significant differences in GM and WM volumes were found between the tinnitus patients and the control group (*p* > 0.05).

### Cerebellar Functional Connectivity in Tinnitus Patients

**Figure 2:**
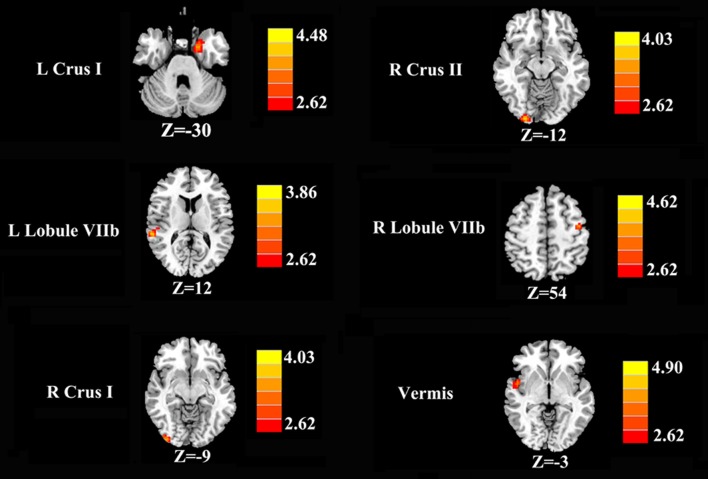
Increased functional connectivity of the different cerebellar seed region of interests (ROIs; bilateral Crus I, bilateral Crus II, bilateral Lobule VIIb and Vermis) in the chronic tinnitus patients compared with the healthy controls. The threshold was set at a *p* < 0.01 (permutation test corrected). Note that the left side corresponds to the right hemisphere.

Increased cerebellar functional connectivity in chronic tinnitus patients was shown in Figure 2 and Table 2. Compared with healthy controls, chronic tinnitus patients showed significantly increased connectivity between the seed region in left Crus I and left PHG; increased connectivity was also observed between the right Crus I and right inferior occipital gyrus (IOG). Relative to controls, tinnitus patients showed significantly enhanced connectivity between the seed region in right Crus II and right IOG. Moreover, in chronic tinnitus patients, the left Lobule VIIb demonstrated enhanced functional connectivity to the right superior temporal gyrus (STG) while the right Lobule VIIb displayed increased connectivity to the left precentral gyrus (PrCG). Finally, compared to the controls, the tinnitus patients exhibited significantly greater connectivity between the seed region in the vermis and right STG. No significant differences were seen when the seed regions were located in the left Crus II and bilateral Lobule VI.

**Table 2:** Increased cerebellar functional connectivity in tinnitus patients compared with healthy controls.

Seed region	Brain region	BA	MNI Coordinates *x, y, z* (mm)	Peak T score	Cluster size
L Crus I	L parahippocampal gyrus	36	−18, 0, −30	3.9336	100
L Crus II	−	−	−	−	−
L Lobule VI	−	−	−	−	−
L Lobule VIIb	R superior temporal gyrus	22	60, −36, 12	3.8120	44
R Crus I	R inferior occipital gyrus	18	36, −93, −9	3.7656	40
R Crus II	R inferior occipital gyrus	18	24, −96, −12	3.9586	71
R Lobule VI	–	–	–	–	–
R Lobule VIIb	L precentral gyrus	6	−42, −9, 54	3.8931	67
Vermis	R superior temporal gyrus	22	51, 3, −3	3.7577	43

*The threshold was set at a p < 0.01, permutation test corrected. BA, Brodmann’s area; MNI, Montreal Neurological Institute; L, left; R, right*.

### Correlation Analysis Results

**Figure 3:**
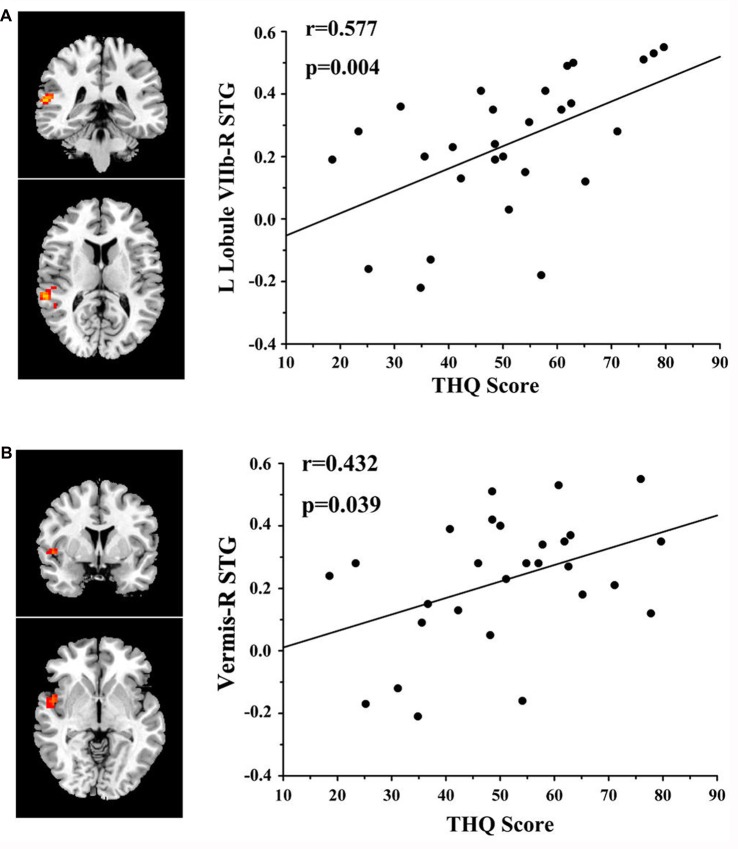
Significant correlations between cerebellar functional connectivity and tinnitus characteristics. **(A)** Positive correlation between the functional connectivity of the left Lobule VIIb to the right superior temporal gyrus (STG) and the tinnitus handicap questionnaires (THQ) score. **(B)** Positive correlation between the functional connectivity of the vermis to the right STG and the THQ score.

In tinnitus patients, the functional connectivity between the left Lobule VIIb and right STG was positively correlated with the THQ score (*r* = 0.577, *p* = 0.004; Figure 3). Furthermore, the functional connectivity between the cerebellar vermis and right STG was also positively correlated with the THQ score (*r* = 0.432, *p* = 0.039). These correlations had been corrected for age, sex, education, GM volume and average hearing thresholds. None of the other regions of increased functional connectivity were correlated with THQ score or tinnitus duration. None of the regions of increased functional connectivity were correlated with SAS or SDS score. Nevertheless, no significant correlations persisted after Bonferroni correction.

## Discussion

This study identified for the first time that enhanced resting-state cerebellar-cerebral functional connectivity is linked to the perception of tinnitus and tinnitus distress. The chronic tinnitus patients experienced enhanced resting-state cerebellar functional connectivity to the auditory cortex and to non-auditory brain regions, such as the limbic system and occipital cortex. The cerebellum plays a crucial role in the auditory processing of tinnitus, in that it may impact the path of audition as a relay node (Petacchi et al., 2005) or may change the virtual noise with a feedback process (Baumann et al., 2015). The increased connectivity between the cerebellum and auditory regions could mediate this role of the cerebellum. In addition, the THQ score was positively correlated with increased cerebellar functional connectivity, which may be another piece of evidence to support the hypothesis that the cerebellum may affect brain networks to produce the unpleasant clinical features of tinnitus, in the form of a modification to the functional connectivity in the brain networks related to tinnitus (Maudoux et al., 2012; Shore et al., 2016). The current results also conform to the hypothesis that the cerebellum, as a crucial node, fails to gate the tinnitus signal, thus producing tinnitus, as posited by Rauschecker et al. (2010).

There are several potential mechanisms by which the cerebellum may be involved in tinnitus. Petacchi et al. (2011) found that the cerebellum is involved as a key region in sensory data acquisition and pitch discrimination. In addition, it has been demonstrated that tinnitus involves network dysfunction (Leaver et al., 2016; Shore et al., 2016). Therefore, the “noises” associated with tinnitus may be the result of a failure to discriminate and encode the different pitches of acoustic sensation, or a mismatch in some other nervous activities (Petacchi et al., 2011). The cerebellum may also perform adaptive signal processing by serving as a comparator of anticipated perception events with received sensory input (Bauer et al., 2013a). A decrease in sensory input may trigger compensatory feed-forward excitation that attempts to normalize input to rostral circuits through increased gain. Thus, the abnormalities in the cerebellar circuitry could lead to inappropriate dynamic modulation of the internal representation of silence (D’Angelo, 2011; Bauer et al., 2013a).

Furthermore, the cerebellum acts as a gain-control mechanism by comparing the afferent input from the cochlea with descending signals from the auditory cortex (Bauer et al., 2013a). Consistent with this view, our results show that chronic tinnitus leads to increased functional connectivity between the auditory cortex and the cerebellum. This gain-control mechanism leads to a highly specific filtering of repetitive unwanted noises, which, as a consequence, do not reach conscious perception in the auditory cortex (Rauschecker et al., 2010). We suggest that when the cerebellar-cerebral functional connectivity is intact, the tinnitus signal is filtered out and cannot be relayed to the auditory cortex. Once this connectivity is disrupted, cancellation of the tinnitus signal will no longer be possible, thus resulting in the perception of tinnitus, followed by long-term reorganization of the auditory cortex to render the tinnitus chronic (Rauschecker et al., 2010). Therefore, the abnormal enhancement of the functional connectivity between the auditory cortex and the cerebellum may be a constituent of the pathological conditions of tinnitus through the failure to filter unpleasant sound in the auditory pathway.

The auditory cortex is thought to be an entrance to the confused tinnitus network, which is related to the PHG region (De Ridder and Vanneste, 2014). Left PHG region showed significantly increased connectivity with the left Crus I in our study. Using the SPECT approach, Laureano et al. (2014) detected that the left PHG had higher rCBF in tinnitus patients compared with healthy controls, suggesting that the limbic system was involved in the disorder of tinnitus. Limbic activity may be associated with an emotional reaction to the experience of phantom sound (Jastreboff and Jastreboff, 2000). In addition, it is thought that the parahippocampal structure is involved in the establishment of the auditory memory of tinnitus by preventing the modification or elimination of hippocampal memory and avoiding habituation (Shulman et al., 1995; Vanneste et al., 2011). The brain will replenish the wrong signal from auditory memory via the parahippocampal area, especially in tinnitus patients with more severe hearing loss (Vanneste and De Ridder, 2016). Moreover, previous resting-state fMRI studies also provided further support linking tinnitus neuropathology with the PHG region (Maudoux et al., 2012; Chen et al., 2015b, 2017c; Leaver et al., 2016).

The occipital cortices that are associated with visual recognition, including the IOG, showed enhanced functional connectivity to the cerebellum in this study. Similar to the auditory cortex, the visual cortex has resting functional connectivity with the cerebellum that is involved in the feedback mechanism of motor control (O’Reilly et al., 2010). Consistent with our result, Chen et al. (2017b) found positive correlation between the tinnitus distress and enhanced inter-hemispheric connectivity in the visual cortex. Moreover, Cate et al. (2009) found that auditory attention could activate peripheral visual cortex. One possible interpretation of the result is that as patients attend to their phantom auditory perception they contemporaneously activate visual regions (Murray et al., 2005). Nonetheless, further studies are needed to reveal the direct relationship between the visual cortex and the cerebellum in tinnitus patients. Furthermore, the strengthened connectivity between the left PrCG and the cerebellum may be a result of the aberrant neuronal activity under the regulation of the cerebellum in the control of movement (O’Reilly et al., 2010). The disrupted connectivity between the cerebellum and the visual and sensorimotor cortices may also be found in other studies (Cacace, 2003; Chen et al., 2016, 2017a), suggesting that tinnitus can be caused directly or modulated by signals from the somatosensory, somatomotor, and visual-motor systems in some individuals. This indicates that tinnitus can be regarded as a consequence of the cross-modal neural interaction of brain networks.

The current study did not detect any GM volume differences between our normal hearing tinnitus patients and matched healthy controls. This finding was, however, consistent with our previous studies (Chen et al., 2014, 2015a,b,c). Although decreased or increased GM volume in several brain regions of tinnitus patients has been reported from previous studies, the changes in GM volume seen in the tinnitus patients of this study were typically correlated with hearing loss, particularly when testing was extended beyond 8 kHz (Leaver et al., 2012; Seydell-Greenwald et al., 2012; Boyen et al., 2013, 2014). Moreover, the heterogeneity of the tinnitus population and the MR analytical method may contribute to the differences in observations between studies. Nonetheless, our results suggest that increased cerebellar functional connectivity can exist prior to major GM volume alterations in tinnitus patients with normal hearing.

Several constraints of the current study must be acknowledged. First, the current study was cross-sectional with a relatively small sample size. It is not appropriate to make direct causal inferences regarding the relationships between increased cerebellar functional connectivity and developing tinnitus characteristics. Therefore, further longitudinal investigations using fMRI experiments would be beneficial to establish the cause-effect relationships. Second, there was an overlap between the cerebellar seed region and the increased functional connectivity. The cross-correlation effects between the different seed regions of cerebellum need to be further analyzed. In addition, multivariate pattern analysis (MVPA) is a data-driven classification technique that can assess the contribution of multiple voxels simultaneously (De Martino et al., 2008), which can be applied to analyze whole-brain functional connectivity patterns of the most discriminative brain areas between the tinnitus and control groups. Thus, the MVPA technique combined with functional connectivity mappings will be applied to strengthen our current results in a future study. In addition, only linear functional connectivity analysis was included in the current study. Some non-linear connectivity methods, such as dynamic causal modeling (DCM; Seghier et al., 2010), are required to indicate the non-linear relationships between the signals among different brain regions in tinnitus. Moreover, different subtypes of tone categories, etiology, severity and other factors that are associated with chronic subjective tinnitus need to be sorted out to prevent the disordered effect of the inconformity of these subgroups. Additionally, we cannot completely prevent participants from hearing some scanner noise, although this study attempted to minimize the noise with earplugs. The existence of scanner noise may make the internal sound of tinnitus less salient, thereby reducing the differences in the cerebellar networks between the tinnitus and control groups. We admit that much of the tinnitus sound might have been masked by the scanner noise. More optimized sampling strategies, such as sparse sampling, will be used to permit highly accelerated acquisitions for improved spatial and temporal resolution fMRI in order to reduce the effect of the scanner noise as much as possible in our future studies (Lustig et al., 2007). Finally, more research is required to obtain structural evidence, such as data from diffusion tensor imaging (DTI), to demonstrate the basis of the functional modulation between the cerebellum and the cerebral cortex.

## Conclusion

In spite of these limitations, our current study identified for the first time enhanced resting-state cerebellar-cerebral functional connectivity that was linked to the perception of tinnitus and tinnitus distress. These findings mainly explicated the crucial role of the cerebellum and the cross-modal neural interaction in tinnitus patients, which may lead to a better understanding of the pathophysiology underlying chronic tinnitus.

## Author Contributions

YF and Y-CC designed the experiment, collected the data, performed the analysis and wrote the article. HL, C-NM, FB, HC and J-JX helped collect the data and perform the analysis. WX and XY contributed to the discussion and manuscript revision.

## Conflict of Interest Statement

The authors declare that the research was conducted in the absence of any commercial or financial relationships that could be construed as a potential conflict of interest.

## Footnotes

^1^http://www.fil.ion.ucl.ac.uk/spm

^2^http://www.restfmri.net

^3^http://dbm.neuro.uni-jena.de/vbm
